# Correlation between CMV Infection and Post-transplantation New-onset Diabetes Mellitus

**Published:** 2016-08-01

**Authors:** I. Dedinská, Ľ. Laca, J. Miklušica, D. Kantárová, P. Galajda, M. Mokáň

**Affiliations:** 1 Surgery Clinic and Transplant Center, University Hospital Martin and Jessenius Faculty of Medicine, Comenius University, Martin, Slovak Republic; 2 *Clinic of Internal Medicine I, University Hospital Martin and Jessenius Faculty of Medicine, Comenius University, Martin, Slovak Republic*

**Keywords:** Diabetes mellitus, Diabetes mellitus, type 2, Diabetes mellitus, type 1, Cytomegalovirus, Transplantation, kidney transplantation, Chemoprevention, Immunosuppressive agents, Immunosuppression

## Abstract

**Background:**

New-onset diabetes mellitus after transplantation (NODAT) is a well-known complication of transplantation.

**Objective:**

To determine the correlation between CMV infection and NODAT.

**Methods:**

Retrospectively, we detected CMV replication (PCR) in every month after renal transplantation in the first 12 months of the procedure in a homogenous group of patients from the immunosuppression point of view.

**Results:**

In 167 patients (64 with NODAT and 103 in the control group), the average amount of CMV viremia was not significantly different between the NODAT and the control group (p=0.929). In the 10^th ^month of transplantation, we recorded a significantly higher CMV viremia in the NODAT group (p<0.0001), however, in the multivariant analysis, the observed statistical difference vanished. The survival of patients and grafts was 12 months after kidney transplantation without any statistically significant difference between the studied groups (p=0.611 and p=0.538, respectively).

**Conclusion:**

CMV is not a risk factor for NODAT.

## INTRODUCTION

New-onset diabetes mellitus after transplantation (NODAT) is a well-known complication of the procedure. Its development is associated with lower graft function and survival. It also reduces long-term patient survival, mainly because of cardiovascular events [1-3]. Kidney transplant recipients who develop NODAT have variably been reported to be at increased risk of fatal and nonfatal cardiovascular events and other adverse outcomes including infection, reduced patient survival, graft rejection, and accelerated graft loss compared with those who do not develop diabetes. Identification of high-risk patients and implementation of measures to reduce the development of NODAT may improve the long-term patient and graft outcome [4]. In 2003, an international expert panel consisting of experts from both the transplant and diabetes fields set forth the international consensus guidelines for the diagnosis and management of NODAT [5, 6]. It was recommended that the definition and diagnosis of NODAT should be based on the definition of diabetes mellitus and impaired glucose tolerance [IGT] described by the World Health Organization (WHO) [6, 7]. The American Diabetes Association (ADA) guidelines for the diagnosis of diabetes mellitus are provided in Table 1 [4].

**Table 1:** ADA diagnostic criteria for diabetes mellitus

ADA diagnostic criteria for diabetes mellitus
Symptoms of diabetes mellitus: polyuria, polydipsia, unexplained weight loss
OR
Fasting blood glucose ≥7 mmol/L
OR
Glycemia in the 2^nd^ hour of oGTT ≥11.1 mmol/L

Cytomegalovirus (CMV) is one of the most important infections in renal transplant recipients [8-12]. Exposure to the virus, as indicated by presence of detectable IgG anti-CMV antibodies in the plasma, increases with age in the general population and is present in more than two-thirds of donors and recipients prior to transplantation [8]. It is therefore, common for the donor and/or recipient to be CMV-positive at the time of transplantation.

CMV can be transmitted from the donor either by blood transfusion or by the transplanted kidney; the concurrent administration of immunosuppressive drugs to prevent rejection further increases the risk of clinically relevant CMV disease, with induction therapy principally being associated with an increased risk of the disease [13, 14]. Therefore, both the recipient and the donor are routinely tested for anti-CMV antibodies prior to transplantation. CMV disease may manifest as a nonspecific febrile syndrome (*e.g.*, fever, leukopenia, and atypical lymphocytosis) or tissue-invasive infections (*e.g.*, hepatitis, pneumonitis, and enteritis). Tissue-invasive CMV disease is defined as CMV disease and CMV detected in tissue with histology, NAT or culture [15]. 

The link between CMV infection and the development of NODAT was first reported in 1985 in a renal transplant recipient [16]. Limited studies suggest that both asymptomatic CMV infection and CMV disease are independent risk factors for the development of NODAT. In a study consisting of 160 consecutive non-diabetic renal transplant recipients who were prospectively monitored for CMV infection during the first three months after transplantation, Hjelmesaeth and colleagues found that asymptomatic CMV infection is associated with a four-fold increase in the risk of new-onset diabetes (adjusted RR: 4.00; p=0.025) [17]. Patients with active CMV infection have a significantly lower median insulin release compared to their CMV-negative counterparts, suggesting that the impaired pancreatic β-cell insulin release may be involved in the pathogenesis of CMV-associated NODAT. It is speculated that CMV-induced release of proinflammatory cytokines may lead to apoptosis and functional disturbances of pancreatic β-cells [18]. Randomized controlled trials have demonstrated that the incidence of CMV disease can be reduced by prophylaxis and pre-emptive therapies in solid-organ transplant recipients [19-21]. 

According to the recommendations of KDIGO, CMV chemoprophylaxis is indicated (except when both the donor and recipient have negative CMV serologies) by applying the oral ganciclovir or valganciclovir for a minimum of three months after kidney transplantation and for six weeks after kidney transplantation in case of T-cell-depleting antibody therapy [15].

In our department, we apply chemoprophylaxis (valganciclovir) in case of seronegative recipient or seropositive donor (R+/D–) 100 days after transplantation. In case of applying antithymocyte globulin, we apply the prophylaxis for six weeks. However, CMV viremia (using PCR) is monitored regularly in all recipients, except for R+/D– where the viremia is monitored as follows: the first six months after transplantation every two weeks, and from the 6^th^ to 12^th^ month after transplantation once per month.

## PATIENTS AND METHODS

**Figure 1:**
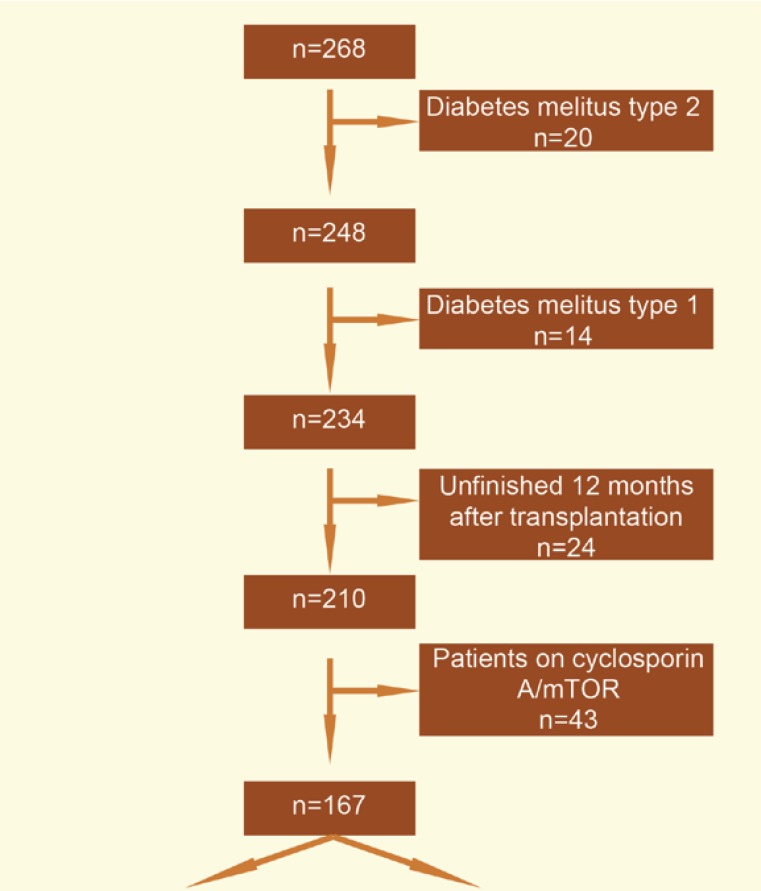
Selection of patients for the analysis

In a retrospective analysis, we monitored CMV viremia as the risk factor for NODAT in a group of patients who underwent primary kidney transplantation from a deceased donor in the Transplantation Center in Martin during 2009–2013. The patients with type 1 or 2 diabetes mellitus and those who underwent kidney transplantation during the last 12 months were excluded from monitoring. The patients who had the mTOR inhibitor or cyclosporin A (Fig 1) in their immunosuppresive regimen were also excluded from monitoring to prevent distortion of results by immunosuppression. In each patient, we recorded the age at the time of transplantation, sex, and the number of HLA mismatches and type of donor (ECD), and identified recipients with risky HLA for NODAT and polycystic kidney diseases. In each patient, we identified CMV viremia by PCR, as customary in our department, two times per month during the first six months from kidney transplantation and once per month from the 6^th^ to 12^th^ month after kidney transplantation. Therefore, during the first six months after transplantation, we had two available values of CMV viremia (copy/mL) for each patient. In the analysis, we used, for each patient, the mean value of the given two measurements. Retrospectively, we identified the patients who had a symptomatic CMV disease during the monitoring period. The patients were divided into two sub-groups according to the development of NODAT within the period—those with NODAT, and a control group. NODAT was diagnosed in a standard way according to the ADA criteria. The groups were compared in terms of development of NODAT and CMV viremia during the entire monitoring period, during the first six months after transplantation and the next following six months after transplantation. We also compared CMV viremia in every month after kidney transplantation. In the end, we compared the function of the graft one year after kidney transplantation (by estimating of glomerular filtration rate (eGFR by applying the CKD-EPI create 2009 formula). We also compared the 12-month survival of the graft (censored for death) and the recipients. Using the correlation coefficient, we identified whether CMV viremia affected the function of the graft 12 months after kidney transplantation. All at risk patients in the monitored group, *i.e.*, the seronegative recipients who received the organ from a seropositive donor and the recipients who received the T-cell-depleting antibody were administered chemoprohylaxis. In case of a seronegative recipient, it was 100 days from kidney transplantation, and in case of the T-cell-depleting antibody, it was six weeks from administration.

We used MedCalc ver 13.1.2. for statistical analysis. *Student’s t* test, χ^2^ test, correlation coefficient, logistic regression analysis, Cox proportional hazard model, and Kaplan-Meier survival anlaysis were used for data analyses. A p value <0.05 was considered statistically significant.

## RESULTS

The studied groups consisted of 64 (38.3%) patients in with NODAT, and 103 (61.7%) patients who served as control. The mean level of tacrolimus (during the 12 months of kidney transplantation) was not significantly different between the studied groups (p=0.559); similarly was the average dose of prednisone/day (p=0.088). The average dose of mycophenolate mofetil/day or mycophenolate sodium was also not different between the groups (p=0.092, and p=0.173, respectively). Therefore, the two studied groups could be considered homogenous in terms of the applied immunosuppression (Table 2). The characteristics of the studied groups are presented in Table 3. The patients with NODAT were significantly older than those in the control group. During the monitoring 12 months, the patients with NODAT received a significantly higher dose of methylprednisolone. However, in multivariate analysis, the dose of methylprednisolone, as the independent risk factor for NODAT, was not identified (Table 4). The mean methylprednisolone dose correlated with the incidence of acute rejection (r=0.261, p=0.011), but CMV replication was not linked to the mean methylprednisolone dose [r=0.163, p=0.116). 

**Table 2:** Comparison of the control group* vs* NODAT in terms of immunosuppression. Values are mean±SD

	Control group n=103	NODAT group n=64	p value
Level of TAC (ng/mL)	4.7±0.9	4.8±1.2	0.5592
Dose of prednisone/day (mg)	8.2±2.3	8.8±2.0	0.0877
Dose of MMF/day (mg)	849.4±264.2	911.7±175.4	0.0919
Dose of mycophenolate sodium/day (mg)	670.7±292	721.9±113	0.1734

TAC: Tacrolimus, MMF: Mycophenolate mofetil

**Table 3:** Characteristics of the studied groups—univariant analysis

	Control groupn=103	NODAT groupn=64	p value
Mean±SD age at the time of transplantation (yrs)	43±12.1	50.5±9.6	<0.001
Males (%)	62.1	59.4	0.863
HLA A30 (%)	2.9	0	0.438
HLA B27 (%)	9.6	10.9	0.994
HLA B42 (%)	1	0	0.834
Average number of HLA mismatches	3.5±1.2	3.7±1.4	0.327
APKD (%)	10.4	17.2	0.284
ECD donor (%)	17.3	21.9	0.593
Pulse therapy by methylprednisolone (%) except for induction	36.4	34.9	0.979
Mean±SD dose of (g) except for induction	2.0±0.7	2.3±0.7	0.009
CMV replication (%)	45.8	45.2	0.929
Average CMV load (copies/mL)	3500	3800	0.976

DM2: type 2 diabetes mellitus, APKD: polycystic kidney disease, ECD: extended criteria donor, CMV: cytomegalovirus

**Table 4:** Characteristics of the studied groups—multivariate analysis

	Hazard Ratio	95% CI
Age at the time of transplantation... (yrs)		
<30	0.307	0.083–1.136
30–39	0.500	0.053–4.752
40–49	0.700	0.429–1.142
50–59	1.138	1.044–1.240
≥60	2.504	1.718–3.649
Pulse therapy by methylprednisolone (yes/no)	2.602	0.742–9.133
Average dose of (g) except for induction	1.103	0.712–1.709

**Figure 2:**
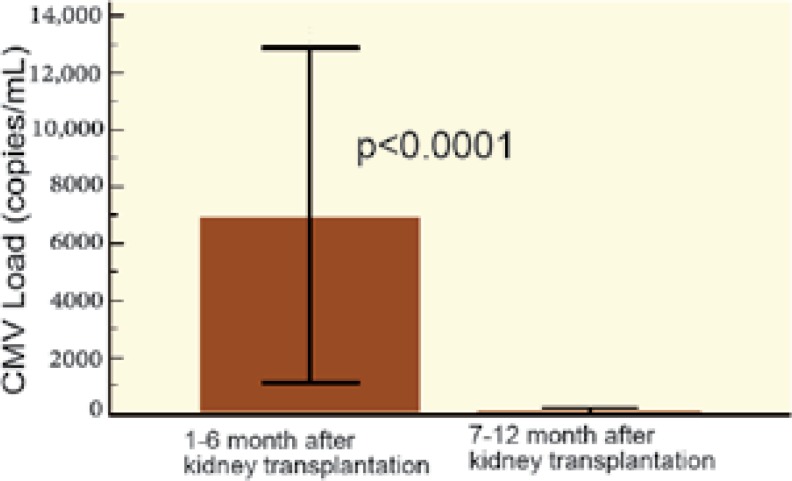
Replication of CMV 1st–6th month* vs* 7^th^–12^th^ month after kidney transplantation* vs* control group

**Figure 3:**
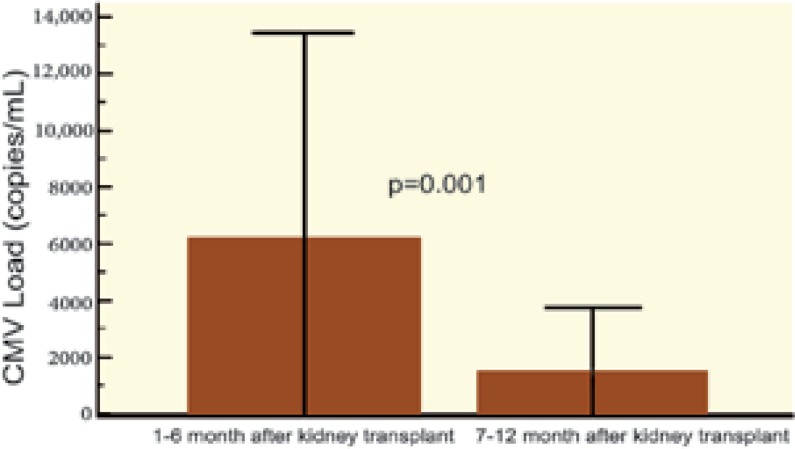
Replication of CMV 1^st^–6^th^ month* vs* 7^th^–12^th^ month after kidney transplantation—NODAT group

**Figure 4:**
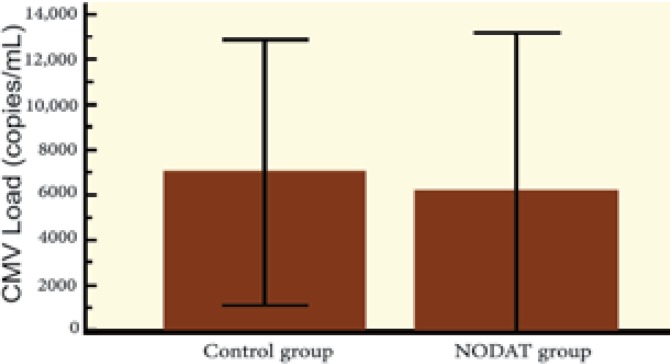
Replication of CMV 1^st^–6^th^ month after kidney transplantation: Control group* vs* NODAT group

**Figure 5:**
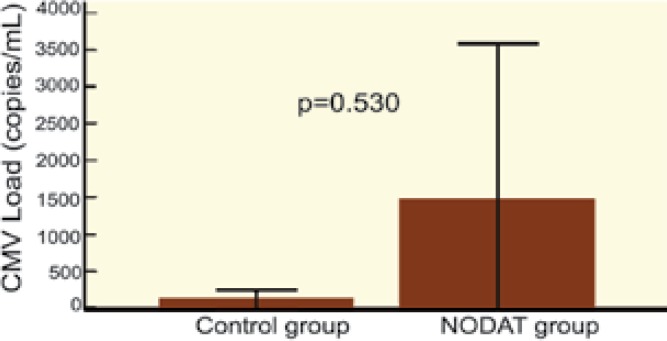
Replication of CMV 7^th^–12^th^ month after kidney transplantation: Control group *vs* NODAT group

The average amount of CMV viremia (copy/mL) was not significantly different between those with and without NODAT. We compared replication of CMV in the first six months of kidney transplantation with replication during the second half-year after transplantation; there was significantly more CMV replication in both groups during the first half-year after transplantation. However, upon comparing the control group with the NODAT group, no difference was observed in CMV replication between the first and the second half-year after kidney transplantation (Figs 2-5).

**Figure 6:**
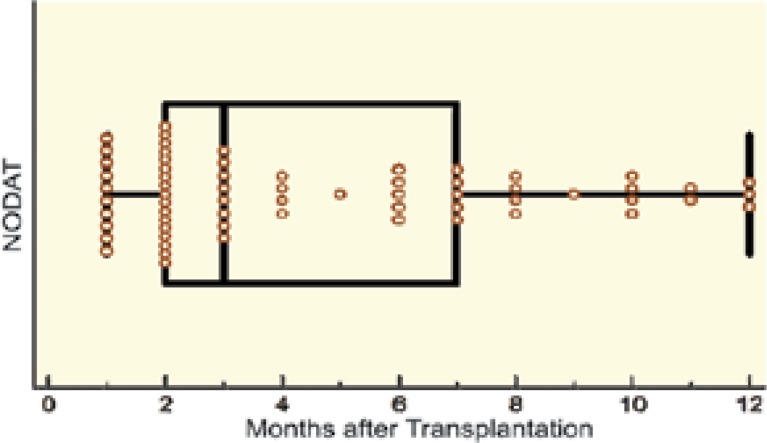
Time of diagnosis of NODAT (months after transplantation

The level of CMV viremia after kidney transplantation is presented in Table 5. In the 10^th^ month after kidney transplantation, we recorded a significantly higher CMV viremiain in the NODAT group. However, the difference was not significant confirmed in multivariate analysis (Tables 5, 6). Therefore, it seems that CMV was of no relevance to development of NODAT during the first 12 months of kidney transplantation. Most (70%) of those who developed NODAT, developed the disease within the first six months of transplantation (p<0.001) (Fig 6).

**Table 5:** CMV replication—individual months after kidney transplantation (univariate analysis

Months after transplantation	Control group (n=103) CMV PCR (copies/mL)	NODAT group (n=64) CMV PCR (copies/mL)	p value
1	1177.1	0	0.357
2	6489.6	24241.9	0.328
3	26346	4975.8	0.308
4	4578.9	6770.9	0.655
5	659.4	601.6	0.901
6	2729.2	270.9	0.220
7	52.1	2233.9	0.214
8	338.5	250	0.840
9	0	41.9	0.086
10	104.2	48256.6	<0.001
11	177.1	16.1	0.467
12	0	48.4	0.438

**Table 6:** CMV replication—individual months after kidney transplantation (multivariate analysis

Months after transplantation	Odds ratio	95% CI
1	1.000	0.684–1.458
2	1.000	1.000–1.000
3	1.000	1.000–1.000
4	1.000	1.000–1.000
5	1.000	1.000–1.000
6	1.000	1.000–1.000
7	1.000	1.000–1.000
8	1.000	0.999–1.000
9	1.021	0.000–24969.894
10	1.000	1.000–1.000
11	0.999	0.323–3.085
12	1.007	0.003–328.380

In the whole group, only 6% of patients developed symptomatic CMV infection. In the NODAT group, 10.9% of patients had symptomatic CMV infection; in the control group, it was 2.9% (p=0.0741).

**Figure 7:**
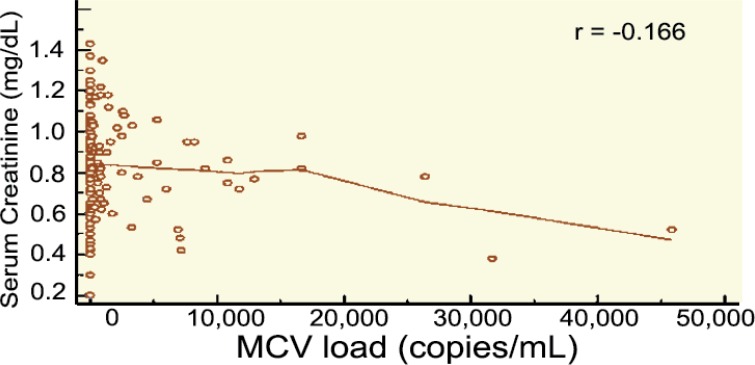
Correlation between CMV and eGFR (CKD-EPI) 12 months after kidney transplantation

The serum creatinine level as well as the eGFR 12 months after transplantation were comparable in both groups (Table 7). A higher CMV load was associated with worse graft function (as determined by eGFR) 12 months after kidney transplantation (Fig 7).

**Table 7:** Comparison of function of the graft (creatinine and eGFR) 12 months after transplantation. Values are mean±SD

	Control group n=103	NODAT group n=64	p value
Creatinine 12 months after transplantation (µmol/L)	139.4±38.1	140.1±43.6	0.914
eGFR 12 months after transplantation (mL/min)	51±14.4	46.8±13.2	0.064

## DISCUSSION

Many risk factors have so far been found to have influence on development of NODAT. In 1985, Lehr, *et al*, reported a case of CMV-induced NODAT in a kidney recipient. Thereafter, the role of CMV infection in development of NODAT has been an area of interest for researchers [22]. Since then, some studies have supported [17, 23] the relationship between CMV infection and NODAT, whilst other studies [24, 25] have failed to prove this association. However, the influence of CMV infection on developing NODAT is still controversial. If the impact of CMV infection on higher incidence of NODAT is proven, initiating prophylaxis against CMV infection after transplantation will be strongly suggested [26]. In a meta-analysis conducted by Einollahi, *et al*, it is shown that the risk of NODAT in kidney transplants with CMV infection is 1.94-fold higher than those without CMV infection [27]. Two studies [28, 29] reported no significant relationship between CMV infection and NODAT; three studies [17, 23, 30] identified CMV infection as a risk factor for NODAT. In addition, Valderhaug and coworkers [31] only found a significant association between CMV infection and NODAT in univariate analysis; the association vanished in multivariate analysis. 

According to the above-mentioned meta-analysis [27], the studies used different criteria to identify CMV infection. Isolation of the CMV virus and detection of viral proteins or nucleic acid are different ways to recognize CMV infection [27]. In addition, active systemic CMV infection can be diagnosed by detection of CMV-DNA in plasma by PCR or by detection of CMV-antigen in leucocytes (*i.e.*, CMV-pp65) [18]. Four of seven studies considered in the analysis did not report the criterion for identification of CMV infection [23, 28-30]. The three remaining studies used different criteria for the diagnosis of CMV infection; Hjelmesaeth, et al [17], defined CMV infection as one or more CMV-pp65 antigen-positive cells per 100,000 leucocytes; Marin and colleagues [25] defined it as >50 infected cells per 200,000 leucocytes using the pp65 assay or isolation of CMV antigenemia or a four-fold increase in the baseline IgG; and Valderhaug, et al [31], diagnosed it by CMV-pp65 antigen in leucocytes or CMV-DNA in plasma. Nonetheless, they did not report any further details. Such a high variance in the criteria and diagnostic methods used can certainly lead to over- or underestimation of CMV infection in the studies. The studies which determine CMV viremia by PCR may explain the relationship between CMV and NODAT.

We found no association between CMV load and development of NODAT. The two studied groups were homogenous in terms of immunosuppresion. The results of our analysis and the low occurrence of symptomatic CMV infection could be, in our opinion, attributed to the intensive monitoring of CMV viremia (PCR) after transplantation (the first six months, CMV viremiais determined every month; in the second half-year, every six weeks). In the at risk patients (seronegative donor and seropositive recipient), we also monitor CMV viremia in the second year after transplantation, every two months. Patients who were treated with T-cell depleting antibody have monitored for CMV viremia every month for three months after the end of therapy. Recipients with increased risk of CMV infection were administered chemoprophylaxis according to the KDIGO recommendations of 2009 [15].

A cohort study conducted by Smedbråten, *et al*, is an extension of the previous study reporting effects of CMV on the graft and patient survival in 471 patients who underwent kidney transplantation between 1994 and 1997. None of the patients received CMV prophylaxis or pre-emptive treatment. CMV infection was an independent risk factor for mortality in multivariate analysis (HR: 1.453, 95% CI: 1.033–2.045) [32]. This observed association between CMV infection and long-term graft and patient outcome may be altered by prophylaxis or pre-emptive CMV therapy. In a study conducted by Kliem, *et al*, prophylactic oral ganciclovir was compared to intravenous pre-emptive CMV therapy [33]. Compared to pre-emptive therapy, prophylaxis was found to be significantly associated with improved 4-year uncensored graft survival, with the greatest benefit observed in the donor+/recipient+ CMV serostatus group. Moreover, when analyzing the death-censored graft survival, the prophylaxis significantly improved graft survival in the donor+/recipient+ CMV serostatus group. Opelz, *et al*, reported from analyses of register data that CMV prophylaxis was significantly associated with improved graft survival both censored and uncensored for death; but, in both cases only in the donor+/recipient– CMV serostatus group [34]. In our study, we identified an association between CMV viremia and function of the graft 12 months after kidney transplantation. With increasing CMV load, eGFR worsened one year after kidney transplantation.

CMV replication after transplantation may contribute to reduced graft function and survival through the associated inflammation and cytokine release [35]. Uncontrolled replication of CMV affects directly or indirectly the recipient [36]. When CMV is reactivated after immunosuppression, it has both direct and indirect effects, such as development of CMV disease, and increased incidence of allograft rejection, respectively [37].

In our study, survival of the patients (censored for death) as well as survival of the graft was numerically worse in the NODAT group, though the difference was not statistically significant. We believe the long-term (>10 years) survival of patients with NODAT is significantly lower than those without NODAT. Intensive glycemic control and early diagnostics and treatment of NODAT, as well as check-up for other risk factors for NODAT can presumably improve the survival of both recipient and grafts. Regular measurement of weight and waist circumference in patients after kidney transplantation leads to identification of at risk patients for NODAT. Screening of the potential risk factors for diabetes mellitus should be done before placing the patient on the waiting list. It is advisable to carry out an oral glucose tolerance test in patients with physiological levels of fasting glycemia [38, 39].
